# Proceedings of the Iowa State Dental Society

**Published:** 1881-07

**Authors:** E. E. Hughes

**Affiliations:** Rec. Sec’y.


### PROCEEDINGS OF THE IOWA STATE DENTAL SOCIETY.

The nineteenth annual meeting of the Iowa State Dental Society was held in Davenport, May 9th, 10th, 11th and 12th, 1881.

The following were elected officers for the ensuing year:

President, Dr. G. W. Fuller, Des Moines; Vice-President, Dr. A. V. Eaton, Anamosa; Corresponding Secretary, Dr. A. O. Hunt, McGregor; Recording Secretary and Treasurer, Dr. E. E. Hughes, Newton.

### COMMITTEES—EXECUTIVE COMMITTEE.

Drs. L. C. Ingersoll, Keokuk; T. A. Hallett, Des Moines, and S. A. Garber, Tipton.

### COMMITTEE ON MEMBERSHIP.

Drs. W. P. Dickinson, Dubuque; J. T. Abbott, Manchester, and R. S. Rathbern, Clinton.

### PUBLICATION COMMITTEE.

Drs. E. E. Hughes, Newton; J. S. Kulp, Muscatine, and L. E. Rogers, Ottumwa.

### COMMITTEE ON DENTAL LEGISLATION.

Drs. I. P. Wilson, Burlington; W. O. Kulp, Davenport, and E. E. Hughes, Newton.

### COMMITTEE ON DENTAL DEPARTMENT IN UNIVERSITY.

Drs. N. H. Tulloss, Iowa City; L. C. Ingersoll, Keokuk, and A. O. Hunt, McGregor.

### COMMITTEE ON REVISION OF BY-LAWS.

Drs. A. O. Hunt, McGregor; I. P. Wilson, Burlington, and L. C. Ingersoll, Keokuk.

### ESSAYISTS FOR 1882.

L. C. Ingersoll, Keokuk; I. P. Wilson, Burlington; J. Hardman, Muscatine; R. S. Rathburn, Clinton; J. R. Townsend, Iowa City; A. O. Hunt, McGregor; C. S. Tulloss, Lyons; E. E. Hughes, Newton.

DELEGATES TO AMERICAN DENTAL ASSOCIATION.

L. C. Ingersoll, Keokuk; I. P. Wilson, Burlington; A. O. Hunt, McGregor; W. O. Kulp, Davenport; M. L. Jackson, Oskaloosa; J. T. Abbott, Manchester.

The next meeting will be held at Des Moines on the first Tuesday in May, 1882. E. E. HUGHES, Rec. Sec'y.

